# Supplementary material for: Elucidating Hexanucleotide Repeat Number and Methylation within the X-Linked Dystonia-Parkinsonism (XDP)-Related SVA Retrotransposon in TAF1 with Nanopore Sequencing
Source: Genes (Basel). 2022 Jan 11;13(1):126. doi: 10.3390/genes13010126 (PMC8775018; doi:10.3390/genes13010126)
Supplement: Supplementary file 1 [file genes-13-00126-s001.zip › genes-1537069-supplementary.pdf]

Supplementary Materials

Table S1: Overview of patients with XDP for variant and repeat detection.

N	**96**
**Percentage of men (%)**	100
**Mean age at examination (SD)**	45.4 (10.2)
**Mean AAO (SD)**	40.7 (8.8)
**Median age at examination (IQR)**	42.5 (37.5)
**Median AAO (IQR)**	39.5 (35. 45)

AAO - age at onset, AAE - age at examination, SD - standard derivation, IQR - interquartile range

Table S2: Primers for the long-range PCR [30].

**Primer**	**Sequence**
XDP-16153 F	5′-GTTCCATTGTGTGGTTGTACCAGCGTTTGTTC-3′
XDP-19345R	5′-CACATGAAAAGATGCCC AACATCATTAGCCATTAG-3′

Table S3: Sequences of the crRNAs for the Cas9-targeted sequencing.

**crRNA**	**Sequence (5' to 3')**
crRNA1 (upstream)	GCACTAAGATTAGGGTCATTAGG
crRNA2 (upstream)	GCTACATAGTCTGAACCAATAGG
crRNA3 (upstream)	AAACTTCCCCCGATCCTGCTTGG
crRNA4 (upstream)	GTCCAGTCTACCAAGTAAACAGG
crRNA5 (downstream)	GACGTAAGTGTGACGACATTGGG
crRNA6 (downstream)	TGACGTAAGTGTGACGACATTGG
crRNA7 (downstream)	ACAGACGTTCACAATCTCCTGGG
crRNA8 (downstream)	GGATACTCTACATACTCTCCAGG

Table S4. Overview of the Nanopore sequencing quality parameters

	Size of target sequence	Mean read quality	N50	Mean coverage
TAF1 SVA PCR (96 barcoded patients)*	3.2kb	15.88	3.38kb	17,645X
TAF1 SVA & flanking regions - short Crispr Cas9 (blood, L-9995, XDP patient)	5.5kb	10.2	4.5kb	514.0X
TAF1 SVA& flanking regions - short Crispr Cas9 (BG, L-9995, XDP patient)		10.0	4.6kb	525.5X
TAF1 SVA & flanking regions - short Crispr Cas9 (CRB, L-9995, XDP patient)		10.1	4.5kb	1226.0X
TAF1 SVA & flanking regions - short Crispr Cas9 (blood, L-14529,control)	2.8kb	10.9	2.0kb	126.9X
TAF1 SVA & flanking regions - long Crispr Cas9 (blood, L-9995, XDP patient)	22kb	12.8	4.7kb	317.2.9X
TAF1 SVA& flanking regions - long Crispr Cas9 (BG, L-9995, XDP patient)		12.3	4.7kb	22.1X
TAF1 SVA & flanking regions - long Crispr Cas9 (CRB, L-9995, XDP patient)		12.3	4.7kb	64.9X
TAF1 SVA & flanking regions - long Crispr Cas9 (blood, L-14529,control)	20kb	13.5	8.8	591.0X

Size of target sequence=Expected size of the reference sequence of the targeted region, Mean read quality=Mean quality Phred score per read (reported by Nanostat v1.5.0), N50=Sequence length of the read with the shortest length within the group of the longest reads that compose 50% of the sequence data (reported by Nanostat v1.5.0), Mean coverage=Mean sequencing depth over the targeted region (reported by Samtools v1.3.1).
*The quality parameters were calculated as an average over the 96 multiplexed samples

Table S5: Overview of CpG methylation of enhancer sites located in the target region

	**Blood (L-7995)**	**Basal Ganglia (L-7995)**	**Cerebellum (L-7995)**	**Blood (L-14529)**
	**Mean MF (SD)**			
**1**	0.93 (0.16)	0.86 (0.14)	0.87 (0.16)	0.90 (0.12)
**2**	0.65 (0.35)	0.46 (0.29)	0.37 (0.43)	0.69 (0.29)
**3**	0.96 (0.05)	0.94 (0.12)	0.95 (0.05)	0.95 (0.08)
**4**	0.96 (0.04)	0.91 (0.09)	0.95 (0.07)	0.83 (0.03)
**5**	0.96 (0.03)	0.95 (0.04)	0.89 (0.05)	0.84 (0.04)
**6**	0.91 (0.06)	0.79 (0.30)	0.81 (0.17)	0.71 (0.16)
**7**	0.95 (0.04)	0.95 (0.04)	0.92 (0.08)	0.81 (0.13)
**8**	NA*	NA*	NA*	NA*
**9**	0.98 (0.02)	0.74 (0.29)	0.88 (0.14)	0.70 (0.28)
**10**	0.88 (0.13)	NA**	0.92 (0.11)	0.75 (0.05)
**11**	0.99 (0.02)	0.99 (0.03)	0.97 (0.07)	0.89 (0.06)
**12**	0.97 (0.07)	0.96 (0.08)	0.99 (0.02)	0.89 (0.08)

MF - methylation frequency, NA - not applicable
*There were no CpG sites within Enhancer 8
**Enhancer 10 did not have sufficient coverage in the basal ganglia-derived DNA sample to be reliable evaluate

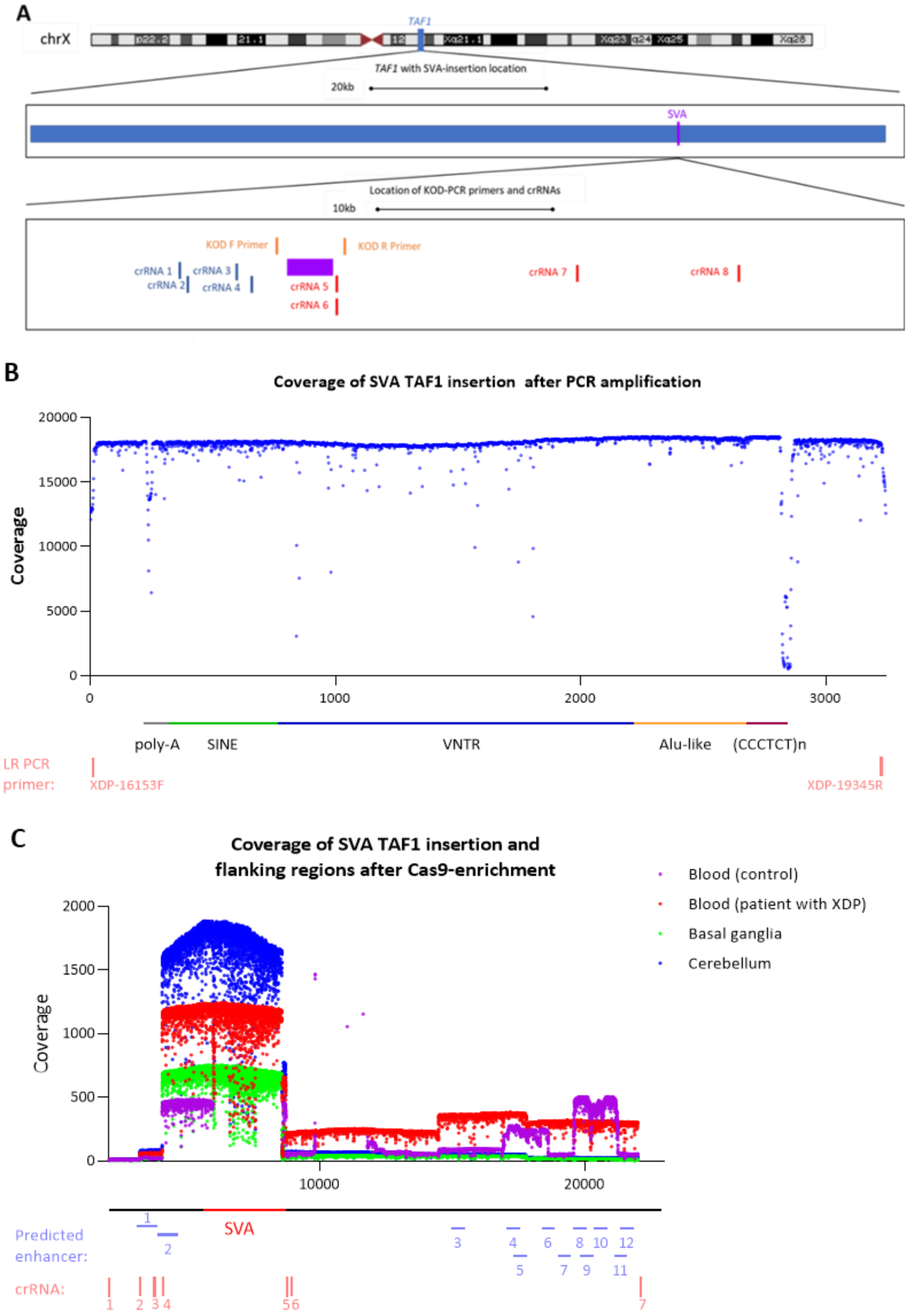

Figure S1. Location of XDP-16153 F and XDP-19345R primers and Cas9 guide RNAs and coverage after PCR or Cas9-targeted enrichment. (A) The bars indicate the location of the TAF1 gene (blue), the SVA (lilac) as well as the primers (orange) and guide RNAs (blue, red) on chromosome X. (B) The plot shows the coverage obtained after the PCR amplification of the TAF1 SVA from a patient with XDP. (C) The plot shows the coverage obtained after the Cas9-guided enrichment of the SVA and flanking regions.

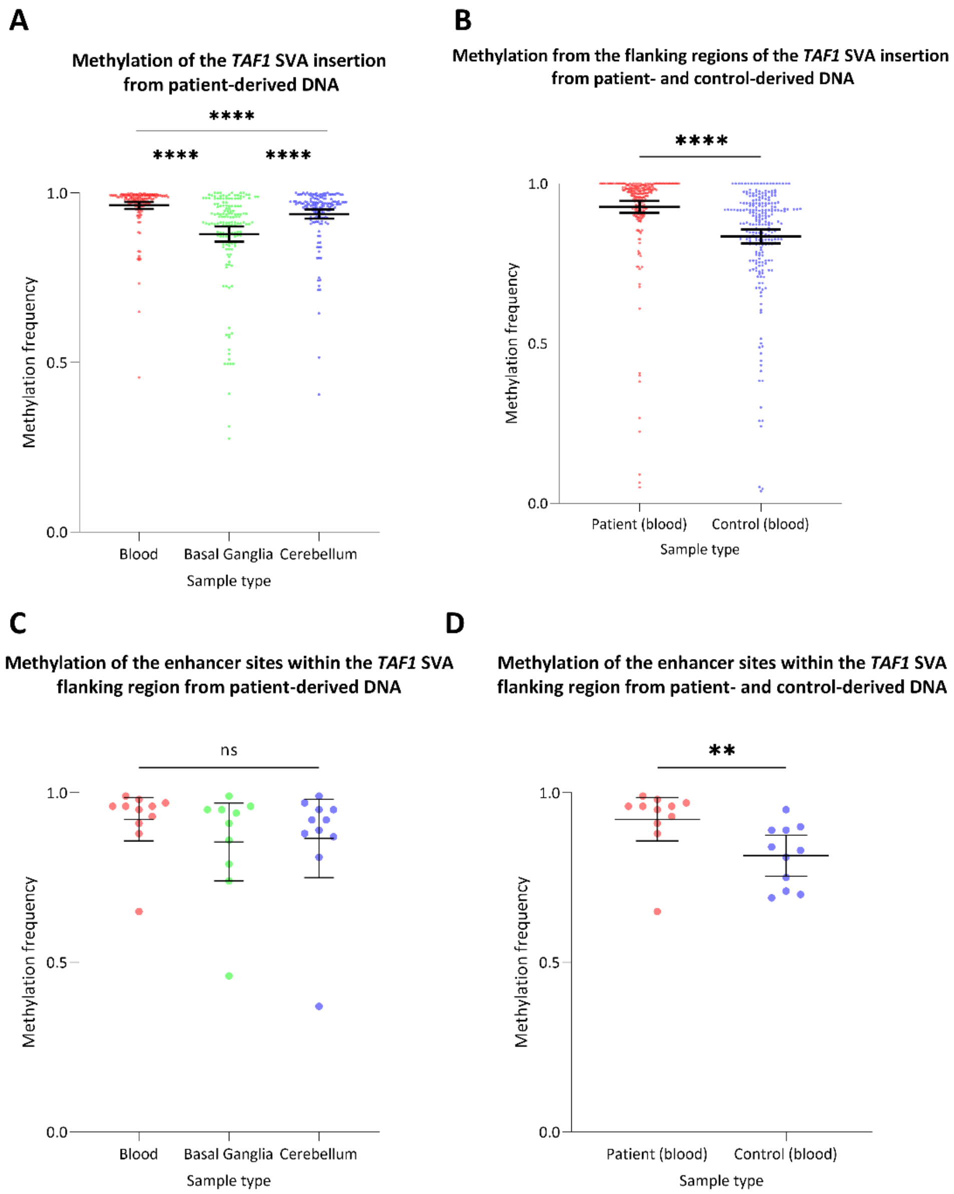

Figure S2. Comparison of the methylation levels of the *TAF1* SVA and flanking regions. (A) The Scatter plot shows the methylation frequency detected in the SVA of a patient with XDP from blood- and brain-derived DNA. (B) The Scatter plot shows the methylation frequency detected in the SVA flanking region of a patient with XDP and a healthy control from blood-derived DNA. (C) The Scatter plot shows the mean methylation frequency detected from predicted enhancer sites located in the SVA flanking region of a patient with XDP from blood- and brain-derived DNA. (D) The Scatter plot shows the mean methylation frequency detected from predicted enhancer sites located in the SVA flanking region of a patient with XDP and a healthy control from blood-derived DNA.
Bars indicate means and 95% confidence interval. The asterisks represent the level of significance (*: p≤.05, **: p≤.01, ***: p≤.001, ****: p≤.0001)., p-value=Mann Whitney U-test performed for pairwise comparison
